# Tumor-targeting bacterial therapy: A potential treatment for oral cancer (Review)

**DOI:** 10.3892/ol.2014.2525

**Published:** 2014-09-11

**Authors:** SAI LIU, XIAOPING XU, XIN ZENG, LONGJIANG LI, QIANMING CHEN, JING LI

**Affiliations:** State Key Laboratory of Oral Diseases, West China College of Stomatology, Sichuan University, Chengdu, Sichuan 610041, P.R. China

**Keywords:** oral cancer therapy, tumor-targeting bacteria, genetic modification, *Streptococcus*

## Abstract

Certain obligate or facultative anaerobic bacteria, which exhibit an inherent ability to colonize solid tumors *in vivo*, may be used in tumor targeting. As genetically manipulated bacteria may actively and specifically penetrate into the tumor tissue, bacterial therapy is becoming a promising approach in the treatment of tumors. However, to the best of our knowledge, no reports have been published thus far regarding the bacterial treatment of oral cancer, one of the most common types of cancer worldwide. In this review, the progress in the understanding of bacterial strategies used in tumor-targeted therapy is discussed and particular bacterial strains that may have great therapeutic potential in oral squamous cell carcinoma (OSCC) tumor-targeted therapy are predicted as determined by previous studies.

## 1. Brief history of tumor-targeting bacterial therapy

The possibility of using bacteria in the treatment of cancer has been recognized for more than a century (1,2). Although it has potential as a novel treatment, the usage of bacteria to target tumors has limitations due to potential biosafety and other deleterious effects, including intrinsic bacterial toxicity, lowered targeting efficiency, genetic instability, and complicated interactions with other therapies (3–7). The original observation of spontaneous tumor regression from concurrent clostridial infection was reported in 1813 (8,9). The first patient with cancer to be purposefully infected with bacteria was possibly cured by German physician Busch in 1868 (2,10). Over 20 years later, in 1890, Coley, a New York physician, found that several patients with inoperable tumors exhibited tumor regression subsequent to being inoculated with *Streptococcus pyogenes*. However, the effect was not as great as to eradicate the disease (11). In 1935, Connell observed tumor regression in advanced cancer during therapy using sterile filtrates from *Clostridium histolyticum*; the author attributed these results to the production of enzymes (12). In 1947, the first study concerning the deliberate injection of *Clostridium* was published (13). Nonetheless, this field was stagnant due to certain drawbacks (14). It was not until 1976, when Morales, Eidinger and Bruce reported successful treatment of bladder cancer with bacillus Calmette-Guérin (BCG), that this field began to increase rapidly (15). Since then, a number of investigative reports, experimental studies and reviews have been published in this area. Due to these efforts, certain attenuated and engineered obligatory anaerobic bacteria, such as *Clostridium*, *Bifidobacterium*, *Salmonella*, *Mycobacterium*, *Bacillus* and *Listeria*, are known to specifically act as antitumor agents, and colonize hypoxic and necrotic regions, which are present in solid tumors while normally absent in other parts of the body.

## 2. Strategies using bacteria to target tumors

**Figure 1:**
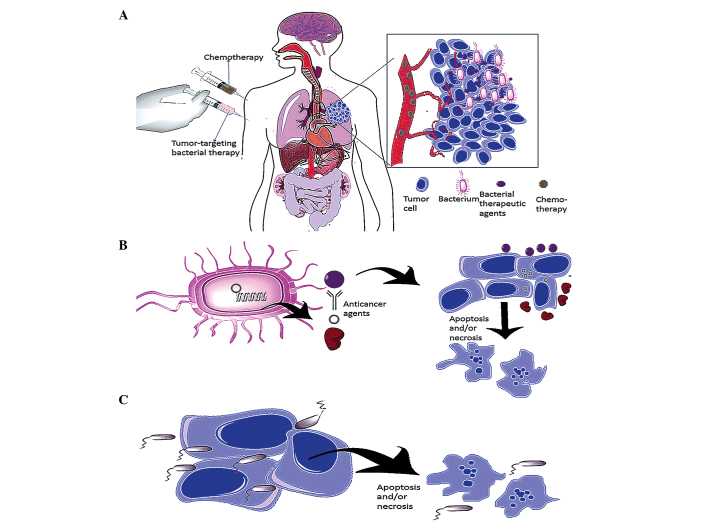
Strategies in tumor-targeting bacterial therapy. (A) Bacteria have adequate tissue penetrating ability. Anaerobic bacteria, which only colonize in areas devoid of oxygen, may actively swim away from the vasculature, penetrate deep into tumor tissue and accumulate following systematic injection (pink syringe), a property traditional chemotherapy (green syringe) does not possess. (B) Delivery of anticancer agents. Bacteria have the ability to manufacture and deliver specific materials, which may be coupled with particular anticancer agents. Engineered bacteria kill cancer cells by expressing proteins that act against tumors (e.g. cytotoxic agents, cytokines, antibodies, cytotoxins, antiangiogenic agents and enzymes that convert the nonfunctional prodrug to an active anticancer drug) and transferring eukaryotic expression vectors into infected cancer cells. (C) Bacteria in oncolytic therapy. Anaerobic bacteria swim into tumor tissue, multiply in the hypoxic/necrotic areas and directly kill tumor cells.

The hypothesis that living bacteria may function as anticancer therapeutic agents was first advanced in the middle of the twentieth century. Due to the obstacles of hypoxia and necrosis, accessing tumor tissue with traditional treatments has proved difficult. However, bacteria may actively migrate away from the vasculature and penetrate deep into tumor tissue and accumulate (Fig. 1A). Three classes of anaerobic and facultative anaerobes have been examined for use in anticancer therapy (16,17): *Bifidobacteria*, facultative intracellular bacteria and strictly anaerobic bacteria.

The ideal criteria for the selection of therapeutic bacteria (18–20) are as follows: Non-toxic to the host; selective for a specific type of tumor; has the ability to penetrate deeply into the tumor where ordinary treatment does not reach; non-immunogenic (does not trigger an immune response immediately but may be cleared by the host); harmless to normal tissue; able to be manipulated easily; and has a drug carrier that may be controlled. In addition to studies of bacteria designed to induce immune responses (21) and mediate antiangiogenesis therapy (22), a recent study has focused on the usage of bacterial products as anticancer agents (23). Three main strategies in bacterial cancer treatment are discussed in this review: i) Bacteria as tumor markers; ii) Bacteria engineered to express anticancer agents (Fig. 1B); and iii) Bacteria for oncolytic therapy (Fig. 1C).

### Bacteria as tumor markers

As replicating anaerobic bacteria are able to selectively target tumors, the use of these bacteria may be an innovative approach for locating tumors that is simple and direct, but practical and effective. Two types of non-bacterial material have served as tumor markers: Viral vectors, including adenovirus, adeno-associated virus, herpes simplex virus (HSV)-1, HSV amplicon, Sindbis, poliovirus replicon and lentivirus/Moloney murine leukemia virus; and non-viral vectors, such as therapeutic DNA, microRNA, short hairpin (sh)RNA, small interfering (si)RNA and oligodeoxynucleotides (ODNs) (24–27). However, anaerobic bacteria are preferable to these other two types of tumor marker due to increased mobility (Table I). Once the marker has been administered, a number of methods may be used to locate the tumor, including bioluminescence, fluorescence and magnetic resonance imaging (MRI), as well as positron emission tomography (6). Bacteria may be detected using light, MRI or positron emission tomography (28,29).

### Bacteria engineered to express anticancer agents

Bacteria exhibit the ability to manufacture and deliver specific materials; these can be artificially coupled to certain anticancer agents (Fig. 1B) (28). The most common current carriers employed in gene therapy are viral vectors, such as retrovirus, adenovirus, viral vaccines, herpes simplex virus and adeno-associated virus. Non-viral delivery systems have been gradually established with the development of technology; currently, the gene therapy field has evolved to encompass not only the delivery of therapeutic DNA, but also of microRNA, shRNA, siRNA and ODNs (20,30,31). However, non-viral gene delivery systems exhibit lower transfection potency, resulting in lowered ability to traverse the various obstacles encountered during treatment (27). Conversely, bacteria have great advantages in the drug carrier field. Two predominant mechanisms have been investigated: The direct expression of antitumor proteins and the transfer of eukaryotic expression vectors into infected cancer cells. In direct expression, four categories of anticancer therapies may be utilized: Proteins with physiological activity against tumors, cytotoxic agents, antiangiogenic agents or enzymes that convert the nonfunctional prodrug to an anticancer drug. In the transfer of eukaryotic expression vectors, gene-silencing shRNAs (32), cytokines and growth factors, and tumor antigens have been investigated (Table II) (7). Furthermore, the number of useful agents is increasing due to new developments in combinatorial synthesis and the advent of metagenomics, which is an unlimited source of novel anticancer bacterial products.

### Bacterial oncolytic therapy

The employment of bacteria in oncolytic therapy is the initial treatment and most direct method to kill tumor cells. Clostridial spores are the main components in oncolytic therapy and have been thoroughly analyzed (22,33,67). Bacterial-based cancer therapies using *Clostridium* spores have the advantage of overcoming the obstacles of hypoxia and necrosis (68). *Clostridium* spp. are strictly anaerobic and only colonize areas devoid of oxygen; therefore, when *Clostridium* spp. are systematically injected into solid tumors, spores germinate and multiply in the hypoxic/necrotic regions. Parker *et al* were the first to demonstrate clostridial oncolysis and tumor regression in mouse tumors by injecting a *Clostridium* spore suspension into transplanted mouse sarcomas 69). However, during follow-up studies, spore treatment with wild-type *Clostridium* was not sufficient to eradicate solid tumors (17,70,71). Thus, genetic engineering and repetitive screens are required to enhance the tumor oncolytic capacity of *Clostridium*. M-55, which was isolated from a non-pathogenic *Clostridium oncolyticum* strain by Carey *et al* (72,73), broke this impasse. Since then, multitudinous recombinant Clostridium strains have been used in tumor treatment. Among these, *C. histolyticium*, *C. tetani*, *C. oncolyticum*, *C. oncolyticum* (sporogenes), *C. beijerinckii* (acetobutylicum) and *C. novyi-NT* have been the most commonly investigated (9,74).

## 3. Advantages and problems of tumor-targeting bacterial therapy

As novel tumor-targeting therapies are introduced, tumor-targeting bacteria have an irreplaceable status due to their unique traits (3). Firstly, it is unsuitable for various types of tumor. Solid tumors are seldom homogeneous; however, almost all tumors have the same microenvironment of low oxygen tension or hypoxia, an environment obligate anaerobes prefer. Furthermore, as bacteria may be easily manipulated, bacteria may be engineered to overcome the limitations that hamper current cancer therapies. In addition, bacteria are highly mobile and actively move away from the vasculature, penetrate deeply and accumulate in tumor tissue. Bacterial therapy achieves adequate tissue penetration, which other treatments, including chemotherapy and radiation, do not (Fig. 1A).

However, certain human trials have shown that the flaws of bacterial therapy cannot be ignored (3–7). As mentioned above, the investigation of bacteria for tumor targeting was stagnant for a long time due to intrinsic bacterial toxicity. In addition, the wild-type bacteria used for therapy, such as *Bifidobacterium longum, Salmonella, Listeria* and *Escherichia coli,* exerted no marked targeting efficiency or oncolytic effect, which reduces the effect of cancer therapy. Furthermore, bacteria exhibit intrinsic genetic instability. Although advanced recombinant DNA technology has rendered it possible to overcome numerous hurdles, bacterial plasmids are not stable and may be lost during bacterial growth.

## 4. Methods and tools used to overcome treatment issues

Several approaches have been employed as attempts to overcome the difficulties mentioned above. Considerable efforts have been recently invested, and synthetic biology techniques are being improved to optimize bacterial therapy and to resolve key challenges. The use of live, attenuated and engineered bacterial strains may abate toxicity (75). The antitumor effects of bacterial treatment were found to be increased by the application of engineered *Clostridium* strains. Saccharolytic *Clostridium, C. novyi-NT* and *E. coli* have been repeatedly screened to become non-toxic with higher tumor colonization (76). *C. oncolyticum* M-55 engineered by Carey *et al* (77), was the first bacterial strain to be genetically manipulated to express an exotic gene, and was used without any side effects. However, the recombinant strains did not function as expected. A non-toxic strain of *Clostridium nocyi* was developed by deleting the virulence gene through heat treatment (78). In addition, a number of genes have been successfully expressed (33–50), which has improved bacterial targeting efficiency and oncolytic effects, as explained previously. A recent study examined and characterized the dynamics of plasmid instability using attenuated strains of *S. typhimurium in vivo*, which produced good results (79).

Bacterial treatment has also achieved gratifying outcomes when administered in combination with other treatments, including antivascular agents, chemotherapeutic drugs, heat shock proteins, heavy metals and radiation. Combined bacteriolytic therapy is a proposed method for cancer treatment that has been relatively successful thus far (68). The combination of particular species with low-dose radiotherapy dampened the tumor immune escape mechanism (79). In addition, *Salmonella* with a modified lipid A (strain VNP20009) was found to be non-toxic and successfully colonized the tumors (81). Although the precise immunological mechanism of BCG therapy remains unclear, increasing numbers of reaction types have been found to be induced by BCG complexes, including infections of urothelial cells or bladder cancer cells, induction of immune responses and induction of antitumor effects (82).

## 5. Analyzing potential OSCC tumor-targeting bacteria groups

Oral cancer, a subtype of head and neck cancer, is defined as cancerous tissue growth located in the oral cavity. Several types of oral cancers have been classified, but 90% of cases worldwide are oral squamous cell carcinoma (OSCC) (83). When the tumor is small enough, a commonly recommended treatment is surgical removal if the outcome would be functionally satisfactory. However, in circumstances in which the tumor is inoperable, radiation therapy with or without chemotherapy is a common treatment option (84). Despite recent advances in diagnosis and therapy, the five-year survival rate of patients with OSCC is only 50% (85). Oral cancer is unusual in conferring a high risk of second primary tumors. This heightened risk may last 5–10 years or occasionally longer (86). Therefore, novel targeting strategies are required to prevent and treat oral cancer. Among the candidate methods of postoperative treatments, tumor-targeting bacterial therapy is expected to have the greatest potential and may even become the main method due to the tumor-targeting specificity.

Specific bacterial species colonize different host locations (87). However, the different roles of the majority of these bacteria have not been determined (88), and may be causal, coincidental or potentially protective. In the human mouth, ~500–1,000 types of bacteria have been detected with various functions; ~110 types constitute the vast majority of oral bacteria (data not shown) (89,90). Three species, *Capnocytophaga gingivalis*, *Pevotella melaninogenica* and *Streptococcus mitis*, have been found to act as diagnostic markers, predicting 80% of oral cancers (86). Although considerable progress has been achieved in elucidating the etiology of oral cancer, the mechanism underlying the association between oral bacteria and oral cancer remains unknown. Further investigation is certainly warranted, but in terms of tumor-targeting therapy, as long as bacteria thrive in OSCC, modern molecular techniques using bacteria may be applied. In addition, artificial modification may further optimize bacteria to meet specific treatment requirements.

**Figure 2:**
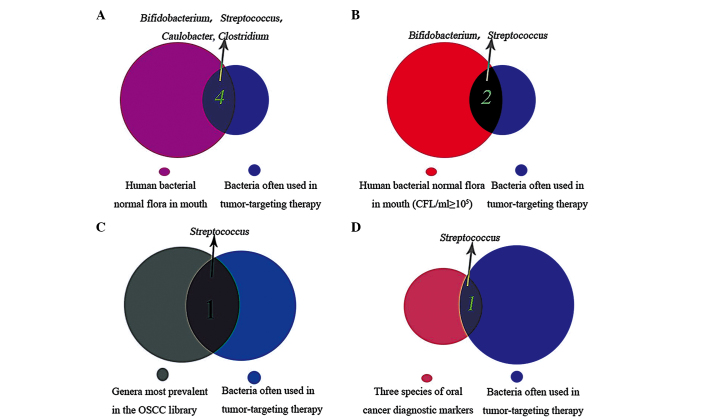
Application potential of different bacteria for oral squamous cell carcinoma (OSCC) tumor-targeted therapy. (A) By retrieving and analyzing studies of human bacterial normal flora in the mouth and the bacteria used in tumor-targeting therapy, *Bifidobacterium*, *Streptococcus*, *Caulobacter* and *Clostridium* were found to have potential as OSCC tumor-targeting therapy bacterial vectors. (B) By retrieving and analyzing studies of human bacterial normal flora [colony-forming units (CFU)/ml≥10^5^] in the mouth and the bacteria used in tumor-targeting therapy, *Bifidobacterium* and *Streptococcus* exhibited greater application potential in OSCC tumor-targeting therapy. (C) Furthermore, by retrieving and analyzing the studies of the genera most prevalent in the OSCC library and the bacteria used in tumor-targeting therapy, *Streptococcus* was found to exhibit the most therapeutic potential as an OSCC tumor-targeted therapeutic bacterial vector. (D) By retrieving and analyzing the three species of oral cancer diagnostic markers and the bacteria used in tumor-targeting therapy, *Streptococcus* exhibited the most therapeutic potential in OSCC tumor-targeted therapy.

Bacteria commonly used in tumor-targeting therapy include *Bifidobacterium*, *Caulobacter*, *Clostridium*, *Escherichia*, *Listeria*, *Proteus*, *Salmonella*, *Streptococcus*, *Mycobacterium* and *Shigella*. As a vector in tumor-targeting treatment, *Salmonella typhimurium* VNP2009, an attenuated mutant of *S. typhimurium*, was first considered due to its significant native toxicity against murine tumors (91). In addition, *S. typhimurium* was analyzed in a first-in-man phase I clinical trial for toxicity and anticancer activity (92). However, *S. typhimurium* is not considered part of the native oral microbiota, which indicates that this species may have a poor OSCC tumor-targeting effect. Six prevalent genera in the OSCC library (93) have been identified: *Streptococcus*, *Gemella*, *Rothia*, *Peptostreptococcus*, *Porphyromonas* and *Lactobacillus* (94). In the present review, the bacteria commonly used in tumor-targeting therapy were compared with the following: Human bacterial flora in the mouth, bacteria with colony-forming units (CFU)/ml ≥10^5^ flora in the human mouth, the genera most prevalent in the OSCC library and three species of oral cancer diagnostic markers (86), respectively. Through this analysis, *Bifidobacterium*, *Streptococcus*, *Caulobacter* and *Clostridium* species were found to have potential for use in OSCC therapy, as these bacteria are part of the normal flora of the mouth, and have previously been used in tumor-targeting therapy (Fig. 2A); *Bifidobacterium* and *Streptococcus* were present at CFU/ml ≥10^5^ commensal flora in the mouth (Fig. 2B). *Streptococcus* may have the most promising OSCC tumor-targeting therapeutic effect, as it is one of the genera most prevalent in the OSCC library and is used as an oral cancer diagnostic marker (Fig. 2C and D).

Promising bacteria used as part of the three main strategies in oral cancer therapy have been discussed in the present review. In order to identify suitable bacteria as diagnostic tools to predict oral cancer, the available information was searched and three of the six most prevalent genera were found in the OSCC library. *C. gingivalis*, *P. melaninogenica* and *S. mitis* predict >80% of oral cancers. In addition, *Candida* spp., which is commonly detected in oral cancer, has been reported to serve as a precancerous diagnostic marker (95). Among the three genera, *S. mitis* may be the best candidate for application as an OSCC tumor-targeting vector due to previous analysis (Fig. 2C and D).

The strategy of employing bacteria engineered to express anticancer agents may be easily used in oral cancer therapy if the correct carriers are selected. *Bifidobacterium*, *Streptococcus* and *Caulobacter* are all suitable, but *Streptococcus* exhibits the most promising therapeutic capacity in this strategy (Fig. 2C and D). Although *Clostridium* is not part of the human bacterial flora of the mouth whose presense is not <10^5^ CFU/ml, it is present in the oral cavity (Fig. 2A), which suggests that *Clostridia* spp. may also be used in OSCC bacterial oncolytic therapy.

However, this is only conjecture according to analysis of the existing data; further experiments are required to verify these hypotheses.

## 6. Conclusion and future perspectives

In the field of cancer treatment, bacterial therapies show great promise, due to the potential tumor-targeting antitumor capability and the ability to deliver therapeutic genes. Currently, one issue in tumor-targeting therapy is selecting the appropriate carrier. The most commonly used carriers are viral vectors, such as retrovirus, adenovirus, viral vaccines, herpes simplex virus and adeno-associated virus. However, the safety, immunogenicity and the limitations of viral vectors are not yet fully understood, and there appears to be no perfect solution for these problems. Thus, as a novel method, bacterial therapy may aid in cancer treatment.

Through this review, *Bifidobacterium*, *Streptococcus*, *Caulobacter* and *Clostridium* spp. were found to be suitable for application in OSCC tumor-targeting therapy. *Streptococcus* exhibited the most promising therapeutic application. Engineered bacteria may further alter mutant bacterial strains to express anticancer agents. Thus, tumor-targeting bacterial therapy has the greatest potential of all candidate methods for oral cancer postoperative treatments.

## Figures and Tables

**Table I:** Materials used as tumor markers.

A, Viral vectors		

Examples	Advantages	Disadvantages
• Adenovirus	• High transfection efficiency	• Generation of immune response
• Adeno-associated virus	• Efficient in initiating gene expression	• Toxicity
• HSV-1		• Possibility of proto-oncogene activation
• HSV amplicon	• Specific targeting	
• Sindbis		
• Poliovirus replicon		• High production cost
• Lentivirus/MoMLV		
		• Limitations in deliverable gene size

B, Non-viral vectors		

Therapeutic DNA, RNAs^a^ and ODNs	Easy to prepare and to scale-up Flexible with regard to the size of the DNA Do not elicit an immune response Less immunogenic Ease of chemical modification Low cost Can be used in different combinations	Low transfection efficiency Less efficient in initiating gene expression
Anaerobic bacteria	Specific targeting High deliverable gene size Motility Can penetrate deep into tumor Easy to manipulate Low cost Environmental sensing Controlled propagation Immunostimulation	Toxicity Genetic instability

a Including microRNAs, short hairpin RNAs and small interfering RNAs.

HSV, herpes simplex virus; MoMLV, Moloney murine leukemia virus; ODNs, oligodeoxynucleotides.

**Table II:** Molecules that may be used as anticancer agents through direct expression by bacteria.

Category	Anticancer molecule	Refs
Cytotoxic agents	Cly A	(34,35)
	FASL	(36)
	TRAIL	(37)
	TNFα	(38,39)
Cytokines	CCL21	(41)
	IL-2	(41,42,43)
	IL-18	(43,44)
	LIGHT	(44,45)
Antigens and antibodies	CtxB-PSA fusion protein	(46)
	CPV-OmpA fusion protein	(47)
	NY-ESO-1 tumor antigen	(48)
	RAF1	(49)
	Single chain HIF1α antibodies	(50)
DNA transfer	Endostatin	(53,57)
	Thrombospondin-1	(54)
	TRAIL and SMAC	(53)
	Stat3	(54,55,57)
	Bcl2	(56,57,58)
	FLT3L	(58)
	GM-CSF	(57)
	IL-12	(58,61)
	AFP	(62)
	VEGFR2	(63)
Enzymes	*E. coli* CD	(64,65)
	HSV-TK	(66)

Cly A (also known as HlyeE), Cytolysin A; FASL, FAS ligand; TRAIL, TNF-related apoptosis-inducing ligand; TNFα, tumor necrosis factor-α; CCL, collagen cross-linking; IL, interleukin; PSA, prostate-specific antigen; CtxB, cholera toxin subunit B; CPV, canine parvovirus; HIF1α, hypoxia-inducible factor 1-alpha; FLT3L, FMS-like tyrosine kinase 3 ligand; GM-CSF, granulocyte/macrophage colony stimulating factor; AFP, α-fetoprotein; VEGFR, vascular endothelial growth factor receptor; CD, cytosine deaminase; HSV-TK, herpes simplex virus thymidine kinase.
